# Compact Sphere-Shaped Airflow Vector Sensor Based on MEMS Differential Pressure Sensors

**DOI:** 10.3390/s22031087

**Published:** 2022-01-30

**Authors:** Kotaro Haneda, Kenei Matsudaira, Ryusuke Noda, Toshiyuki Nakata, Satoshi Suzuki, Hao Liu, Hidetoshi Takahashi

**Affiliations:** 1 Department of Mechanical Engineering, Faculty of Science and Technology, Keio University, 3-14-1 Hiyoshi, Kouhoku-ku, Yokohama 223-8522, Japan; hane_taro@keio.jp (K.H.); kenei.matsudaira@takahashi.mech.keio.ac.jp (K.M.); 2 Department of Aeronautics and Astronautics, Graduate School of Engineering, Kyoto University, Kyoto Daigaku-Katsura, Nishikyo-ku, Kyoto 615-8540, Japan; noda.ryusuke.6a@kyoto-u.ac.jp; 3 Graduate School of Engineering, Chiba University, 1-33 Yayoi-cho, Inage-ku, Chiba 263-8522, Japan; tnakata@chiba-u.jp (T.N.); suzuki-s@chiba-u.jp (S.S.); hliu@faculty.chiba-u.jp (H.L.)

**Keywords:** airflow vector sensor, differential pressure sensor, drone stability

## Abstract

This paper presents an airflow vector sensor for drones. Drones are expected to play a role in various industrial fields. However, the further improvement of flight stability is a significant issue. In particular, compact drones are more affected by wind during flight. Thus, it is desirable to detect air current directly by an airflow sensor and feedback to the control. In the case of a drone in flight, the sensor should detect wind velocity and direction, particularly in the horizontal direction, for a sudden crosswind. In addition, the sensor must also be small, light, and highly sensitive. Here, we propose a compact spherical airflow sensor for drones. Three highly sensitive microelectromechanical system (MEMS) differential pressure (DP) sensor chips were built in the spherical housing as the sensor elements. The 2D wind direction and velocity can be measured from these sensor elements. The fabricated airflow sensor was attached to a small toy drone. It was demonstrated that the sensor provided an output corresponding to the wind velocity and direction when horizontal wind was applied via a fan while the drone was flying. The experimental results demonstrate that the proposed sensor will be helpful for directly measuring the air current for a drone in flight.

## 1. Introduction

In recent years, drones, in the class of unmanned airplanes, have been developed for many applications; for example, they are expected to deliver medicine, vaccines, and other medical devices [1,2]. In the agricultural field, smart agriculture initiatives are underway to monitor farm conditions spatially [3]. Additionally, drones are suitable for inspecting infrastructure facilities where it is difficult for people to enter [4,5]. However, there are potential risks that drones can fall or be blown off course by sudden wind because of their light weight and small size. For flight control, a global positioning system (GPS) and inertial measurement unit (IMU) are generally equipped on drones. However, these sensors do not directly measure the air current that causes loss of body balance. Providing that the wind velocity and direction are directly known in real time, the robustness and stability can be improved in flight control.

Currently, there are various types of airflow sensors in use. A wind turbine-type sensor measures wind velocity from the rotation speed of a propeller and wind direction from the blades that receive the wind [6]. Wind velocity is directly measured as a force; however, it is not suitable for miniaturization because of the sensitivity threshold due to mechanical friction. A hair-cell type sensor also directly measures wind velocity and direction [7]. Since the hair part is directly exposed to wind, it is fragile and not suitable for outdoor use where many objects are subjected to wind forces. An ultrasonic-type sensor measures wind velocity and direction from the propagation time variation between an ultrasonic wave transmitter and receiver [8,9]. The sensor achieves high accuracy; however, it is difficult to make it more compact and less expensive because the measurement system is complicated. A hot-wire type sensor measures wind velocity and direction with high sensitivity by resistance changes due to fluctuations in ambient heat [10,11]. The sensor output is affected by the temperature and humidity of the measurement environment. This characteristic is not suitable for drones flying in open air.

Moreover, Pitot tubes that are generally attached to the front tip of an airplane to measure the relative airflow velocity in the flight direction have been widely used in various fields [12]. The sensor element directly detects the airflow from the DP between two inlets, namely, the dynamic pressure inlet and static pressure inlet, so that the sensor does not respond to other environmental changes in principle. Thus, it is expected that the same detection principle can be applied to an airflow sensor for drones because a drone is also categorized as an airplane. In the case of attaching an airflow sensor to a drone, the required specifications are different from those of airplanes. First, the sensitivity should be higher in the low airflow speed range because the maximum flight speed is approximately 20 m/s, much slower than that of airplanes. Second, drones are smaller and lighter than airplanes, so the sensor should also be more compact and lightweight. Third, drones can fly in all directions, unlike airplanes. Thus, the sensor should measure not only airflow velocity but also airflow direction. In summary, the airflow sensor is required to realize at least the above three specifications to be onboard on drones.

Airflow sensors that adopt the DP principle, which is similar to that of a Pitot tube, have been developed. The sensor shape is a cylindrical or spherical structure so that the wind velocity and direction are measured from the DPs at multiple inlets in the surface [13,14,15,16,17,18]. Although it was thought that miniaturization was difficult due to the complicated housing shape, a compact 2D airflow sensor was developed by placing a thermal sensor element inside [19,20,21,22]. Similarly, it is expected that an airflow sensor that fulfills the required specifications for a drone is realized by minute built-in mechanical DP sensors inside the housing [17,23].

**Figure 1:**
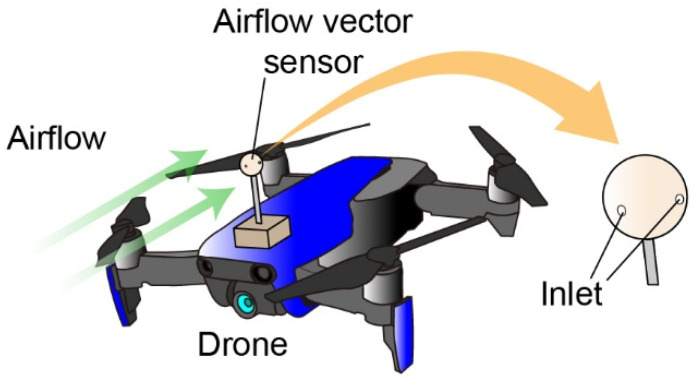
Concept of the proposed airflow vector sensor for a drone.

Here, we propose an airflow vector sensor using MEMS mechanical DP sensors, as shown in Figure 1. The MEMS sensor chip is small enough to be built in a compact sphere-shaped housing. Additionally, high sensitivity is realized by using a piezoresistive cantilever as the sensing element. Multiple inlets are three-dimensionally formed on the sides of the spherical housing. One DP sensor element and one pair of inlets comprise one sensor component. The sensor structure is designed with three sensor components to redundantly measure the 2D wind velocity and direction because of the nonsinusoidal pressure distribution on a spherical surface. In this study, we designed and developed an airflow vector sensor to satisfy the sensor requirements for a small drone. The fabricated sensor was calibrated to wind velocity and direction using regression models. The sensor was then mounted on a drone for airflow measurements.

## 2. Design and Principle

### 2.1. Sensor Design

**Figure 2:**
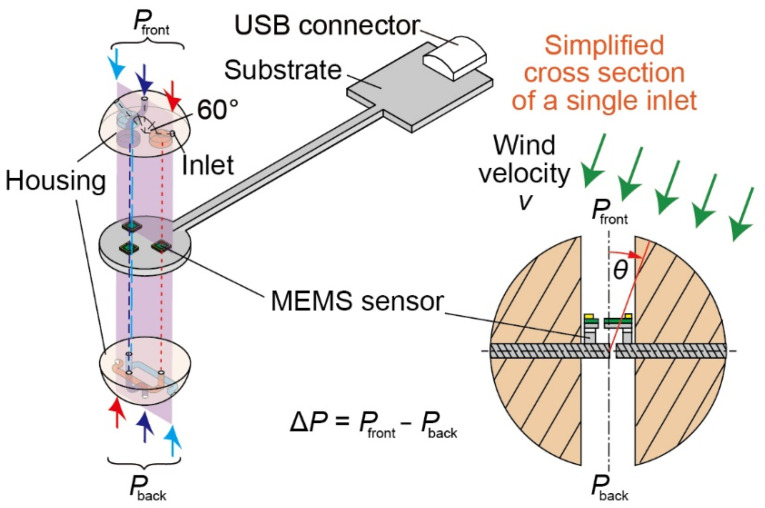
Schematic diagram of the airflow vector sensor component. The sensor is composed of a substrate with three MEMS DP sensor chips and spherical housings with airflow channels. Each MEMS DP sensor measures the DP between the corresponding two channels.

According to the strict sensor requirements in this study, a small drone weighing less than 200 g is selected as the target. Therefore, it is defined that the sensor weight should be less than 10 g, which is 5% of the drone’s weight. In addition, the measurable range and resolution are defined as ~10 m/s and 1 m/s, respectively. Moreover, to avoid the turbulence effect around the propellers, we assume that the sensor is mounted above the fuselage with an approximately 100 mm long supporting shaft. The proposed sensor is designed to be less than 20 mm in diameter and a few grams in total weight to fulfill the requirement. Figure 2 shows the conceptual diagram of the proposed airflow vector sensor with a shaft component. The sensor consists of a pair of hemispherical housings and a substrate with three MEMS DP sensors; the pair of housings sandwich the substrate, forming the spherical shape of the sensor structure. There are air inlets on the spherical surface, while the MEMS DP sensors separate the channel lines between the corresponding air inlets of the upper and lower housings. Then, the DP sensor detects the DP between the two inlets. Each pair of inlets in the upper and lower housings is positioned point-symmetrically to the sphere’s center; three pairs of inlets are arranged. The angle between the adjacent inlets in the same plane is set to 60°, as shown in Figure 2. The DP of each pair does not exhibit a perfect sinusoidal response to the inflow angle. Thus, the redundancy is provided by three sensor elements so that the 2D wind velocity and direction are calculated from the sensor response.

### 2.2. Airflow Detection Principle

The detection principle of the proposed sensor is based on the conversion of DP to wind velocity. Assuming that one pair of inlets of the sphere housing is placed in the front and back of the airflow direction, i.e., *θ* = 0° in the cross-sectional view in Figure 2, a DP is generated between the inlets. Then, the DP MEMS DP sensor inside the channel detects the airflow. According to the theoretical pressure distribution on the spherical surface, the DP Δ*P* is proportional to the square of the wind velocity *v* if the direction *θ* is the same. On the other hand, the DP Δ*P* and wind direction *θ* relationship is derived as a sinusoidal wave in an ideal flow field if the velocity *v* is the same [24]. However, when the sensor-sized spherical structure is placed in an actual airflow, the flow separates or a vortex is generated behind the sensor. The Reynolds number of the sensor structure is approximately 1.0 × 10^4^ at 10 m/s, which is the maximum wind velocity range, resulting in laminar flow. In laminar flow around such a Reynolds number, it is known that nonmonotonic behavior appears in the wind direction range of 70° to 90° in pressure distribution so that a nonmonotonic relationship between the DP and wind direction is observed from 60° to 120° [24].

Wind velocity and direction are not perfectly independent because the response to angle depends on the Reynolds number. Thus, three pairs of inlets are designed at 60° intervals so that each pair is responsible for DP in a monotonic range. Then, their responses are combined to measure the omnidirectional wind direction with redundancy. Since there are three pairs of inlets, three sensor elements are built in the housing. Measuring the DP at the three locations simultaneously for the 2D wind velocity and direction is expected to improve accuracy due to redundancy.

## 3. Fabrication and Assembly

### 3.1. MEMS Sensor Chip and Circuit Design

**Figure 3:**
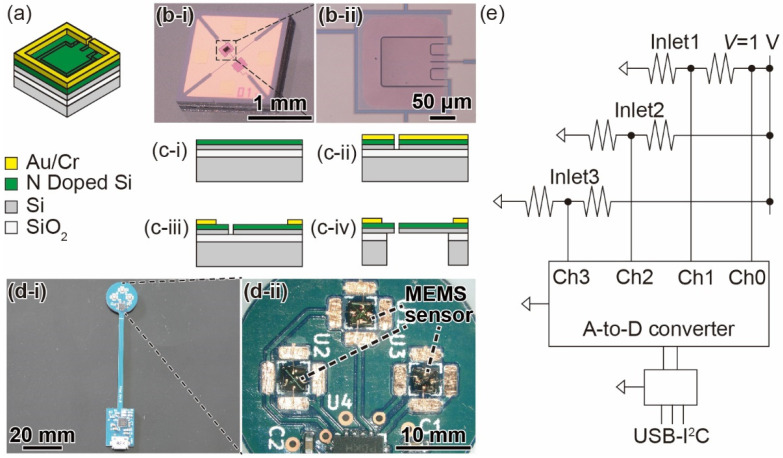
(**a**) Schematic diagram of the piezoresistive cantilever. (**b**) Photographs of (**i**) the MEMS DP sensor chip and (**ii**) the piezoresistive cantilever. (**c**) Fabrication process of the DP sensor. (**d**) Photograph of (**i**) overall and (**ii**) close-up of the substrate with the MEMS DP sensor chips. (**e**) Schematic image of the circuit.

Figure 3a,b shows a conceptual diagram and photographs of the fabricated piezoresistive cantilever-type MEMS DP sensor. The sizes of the MEMS DP sensor and piezoresistive cantilever are 1.5 mm × 1.5 mm × 0.25 mm and 100 µm × 80 µm × 0.2 µm, respectively [25]. When a DP is applied between the upper and lower surfaces of the cantilever, the cantilever bends vertically. The cantilever surface strain changes the resistance of the piezoresistor, which is formed on the cantilever surface. Thus, the DP is measured by detecting resistance changes, which is the basic principle of the resistance change sensing elements. Since the gap surrounding the cantilever is as narrow as 1 μm, there is little air leakage. The MEMS DP sensor chip has two cantilevers: one is used for DP sensing, and the other is used for temperature compensation. The temperature-compensating cantilever does not respond to pressure changes because the handle Si layer is not etched in the fabrication process. Thus, the temperature drift is canceled in DP measurement via a two-gauge method with the two cantilevers. Figure 3c shows the fabrication process for the piezoresistive cantilever [26]. The cantilever is formed on a silicon-on-insulator (SOI) wafer. First, an N-type piezoresistive layer is formed on the device Si layer (Figure 3(c-i)). Then, an Au/Cr layer is formed on the piezoresistive layer, and the cantilever shape is patterned. Next, the device Si layer is etched using inductively coupled plasma reactive ion etching (ICP-RIE) (Figure 3(c-ii)). Then, the Au/Cr layer is etched again (Figure 3(c-iii)). Finally, the handle Si layer and SiO_2_ layer are removed (Figure 3(c-iv)). Both the initial resistances of the sensing and temperature compensation cantilevers are approximately 3 kΩ.

Figure 3(d-i) shows a photograph of the substrate for attaching the MEMS DP sensors. The overall length is 85 mm, and the size of the circular part is *φ*16 mm. The thickness of the substrate is 1.6 mm. The circular part is the housing attachment position, and there are three areas to place the DP sensor chips. The attachment areas have a through-hole 0.45 mm in diameter where the sensing cantilever is designed to overlap. Figure 3e shows a circuit diagram of the substrate with the DP sensor elements. The circuit consists of three DP sensors, a voltage reference chip ISL60002BIH310Z (Renesas Electronics), an analog-to-digital converter chip (ADC) ADS122C04IRTE (Texas Instruments), and a USB-I^2^C interface bridge chip CP2112 (Silicon Labs) [27]. The voltage reference applies a base voltage of 1 V to the bridge circuit of the three pairs of cantilevers. The resistance on the GND side is set to be the sensing cantilever. The bridge circuit converts the resistance change in the sensing cantilever into a voltage change. The output voltages are connected to the ADC, which is close to the MEMS DP sensors to reduce electrical noise. The measurement data are obtained with a PC via an I^2^C bus.

### 3.2. Sensor Housing

**Figure 4:**
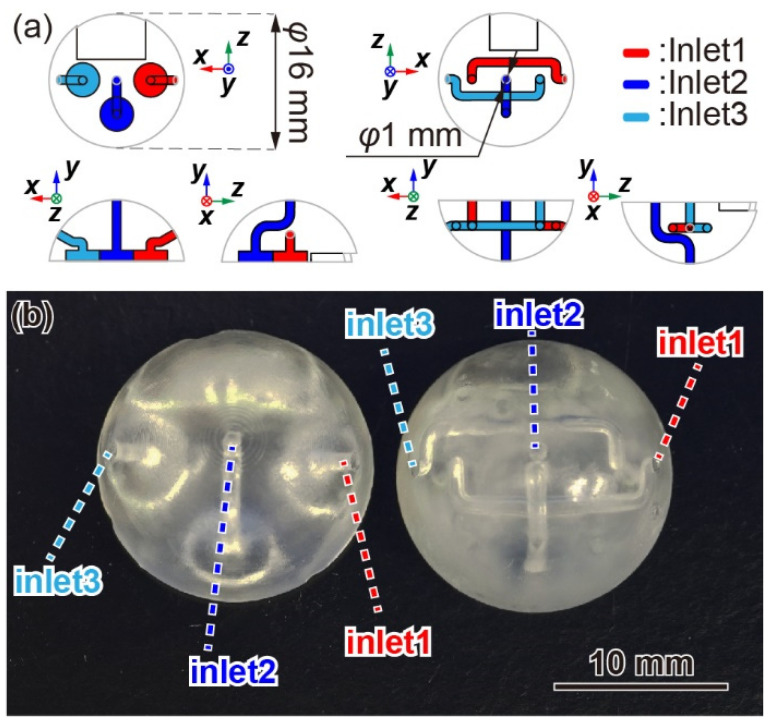
(**a**) Design of the sensor housing with inlets and airflow channel. (**b**) Photograph of the housing fabricated by a 3D printer.

Figure 4a,b shows the design structure and photograph of the fabricated sensor housings. The housings were designed to be 16 mm diameter hemispheres fabricated by a 3D printer (Form 3, Formlabs). The hemisphere bottom face was trimmed by 0.8 mm, considering the substrate thickness. The inlets on the hemispherical surface were designed to be 1 mm in diameter and relatively small on the housing surface area. The right-side inlet was defined as inlet 1, the middle inlet as inlet 2, and the left-side inlet as inlet 3, corresponding to red, blue, and light-blue colors, respectively, in Figure 4a. The channel line in inlet 2 is symmetrical for the upper and lower housings. The channel lines in inlet 1 and inlet 3 are formed to prevent interference with other lines. On the plane of the upper housing, there are three circular holes of *φ*4 mm × 1.2 mm, which are connected to each corresponding channel line to avoid interference with the sensor chips. Additionally, some holes are also formed on each housing’s flat surface to prevent the capacitors and amplifiers from interfering.

### 3.3. Sensor Assembly

**Figure 5:**
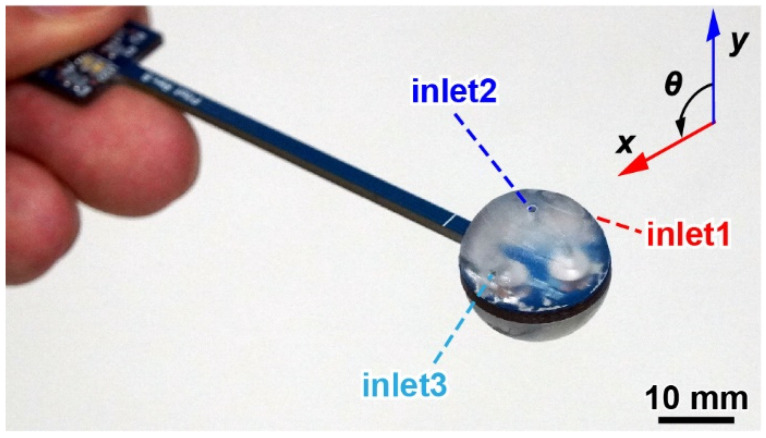
Photograph of the fabricated sphere-shaped airflow vector sensor.

We assembled an airflow sensor using three MEMS DP sensors, one substrate, and a pair of housings. First, the DP sensors were attached to the substrate. At this time, the through-hole and sensing cantilever had to overlap in the same position. Then, the sensors were wire bonded to the substrate for electrical connection. Second, adhesive was applied to the flat part of the housing. Then, the housing was attached to the circular part of the substrate. Figure 5 shows the assembled airflow sensor. The wind direction angle is defined as 0° with respect to the direction of inlet 2 of the upper housings, and the rotation to inlet 3 is the positive direction. The total resulting weight was 4.4 g.

## 4. Experiment and Results

### 4.1. Differential Pressure Calibration

**Figure 6:**
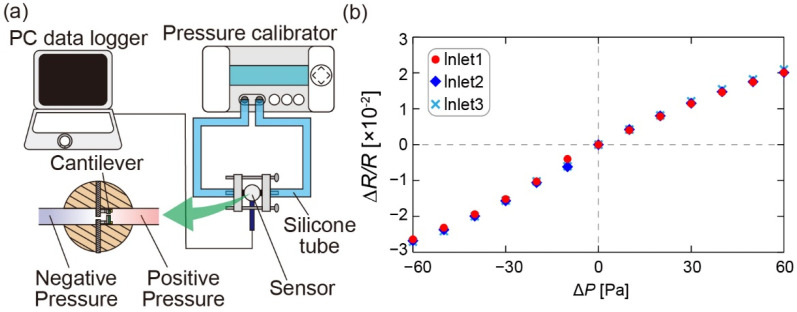
(**a**) Concept of the experimental setup for pressure calibration of the MEMS DP sensor chip built inside the sensor. (**b**) Relationship between the fractional resistance changes and the DP.

We conducted DP calibration with the three inlets of the fabricated sensor. Figure 6 a shows a schematic diagram of the experimental setup. A pressure calibrator (KAL200, Halstrup-walcher GmbH, Kirchzarten, Germany) applied DP to the DP sensor of each pair of inlets through a jig and silicone tube. The output voltage was measured via a PC. In each pair of inlets, DP was applied from −60 Pa to +60 Pa in 10 Pa increments. This DP range approximately corresponds to the airflow velocity range of 10 m/s, as determined via Bernoulli’s equation. The fractional resistance changes in each sensor element were calculated from the output voltage using the equation Δ*R/R* = 4Δ*V* [25]. The relationship between the fractional resistance changes and DP in each inlet is shown in Figure 6b. The responses were proportional to the DP and similar to each other in the three inlets.

### 4.2. Wind Tunnel Test

**Figure 7:**
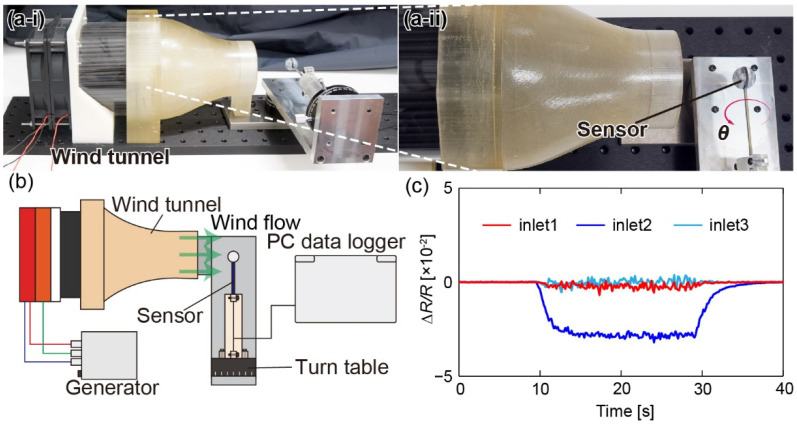
(**a**) Photograph of (**i**) overall and (**ii**) close-up, and (**b**) concept of the experimental setup to measure the sensor response against airflow. (**c**) Responses of the DP sensors against airflow when the wind velocity and direction are 10 m/s and 0°, respectively.

We conducted a wind tunnel test using the airflow sensor. Figure 7a,b shows a schematic diagram and photographs of the experimental setup, respectively. The sensor was placed at the outlet of a compact wind tunnel controlled by a DC fan. The sensor was attached to a two-degree-of-freedom (2-DOF) rotating stage so that the wind direction was varied. The sensor output voltage was measured via a PC. Airflow was applied from 2–10 m/s with a 1 m/s interval, and the rotation angle was changed from −180° to +180° with 15° intervals. In advance, the wind velocity was calibrated by a hot-wire anemometer (Climomaster, Kanomax, Osaka, Japan). The measurements were performed for 10 s with no wind, 20 s with the fan turned on, and 10 s with the fan turned off. Figure 7c shows an experimental result with a wind velocity of 10 m/s and a direction of 0°. The responses of the three DP sensors were recorded simultaneously. The fractional resistance changes in inlet 2 were the largest among the three sensors.

**Figure 8:**
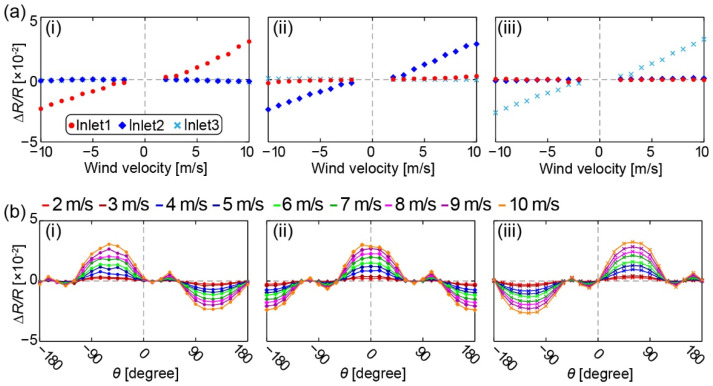
(**a**) Relationship between the wind velocity and the fractional resistance changes in three DP sensors when the corresponding inlet is on the axis of the wind tunnel: (**i**) inlet 1, (**ii**) inlet 2, and (**iii**) inlet 3. (**b**) Relationship between the wind direction and the fractional resistance changes for each inlet.

Figure 8a shows the relationship between the fractional resistance changes and the wind velocity when each inlet pair is parallel to the wind direction. Figure 8a(i–iii) correspond to inlets 1, 2, and 3; the wind direction angles are (i) −60°, (ii) 0°, and (iii) +60°, respectively. The DP sensor of the corresponding inlet pair shows the largest response in each graph, while the DP sensors of the other two inlet pairs show little response. The responses of the three sensor components are similar to each other. For all three DP sensors, the fractional resistance changes, and the wind velocity has a linear relationship over 3 m/s in absolute value. In the range less than 3 m/s in absolute value, the relationship was relatively complicated. Figure 8b shows the relationship between the wind direction and the fractional resistance changes for each of the three inlets. Nonmonotonic behavior was observed in each inlet: −180~−120° and 0~+60° for inlet 1, −120~−60° and +60~+120° for inlet 2, and −60~0° and +120~+180° for inlet 3. In the other angle ranges, sinusoidal-like responses were obtained.

Additionally, the sensor responsivity to wind flow was evaluated. To apply step-responsive airflow to the sensor, we conducted a simple procedure, as described below. The wind tunnel outlet was closed by a flat board, and then the outlet was rapidly manually opened. The wind velocity was set at 5 m/s, and inlet 3 was set on the axis of the wind tunnel. The experimental result is shown in Figure S1. The transition time was found to be approximately 50 ms, indicating a relatively quick response.

### 4.3. Conversion to Wind Velocity and Angle

Regression models were built to convert the output voltages obtained from the three DP sensors into wind velocity and direction. A fifth-order multiple multinomial regression was utilized to construct the models. The inputs and outputs of the models were *V*_1_, *V*_2_, and *V*_3_ and *V*·cos *θ* and *V*·sin *θ*, respectively. The models for each output were built separately. For the ground truth (GT), the data shown in Figure 8 and (*V*_1_, *V*_2_, *V*_3_) = (0, 0, 0) were used for 2–10 m/s and 0 m/s. Each wind velocity data point contains different wind direction data from 0 to 345° in 15° increments. The dataset includes 1339 data points. This model is described by the following equation:

(1) $$\begin{array}{l} y=c_{0}+c_{1}V_{1}+c_{2}V_{2}+c_{3}V_{3}+c_{4}V_{1}^{2}+c_{5}V_{1}V_{2}+c_{6}V_{1}V_{3}+c_{7}V_{2}^{2}+c_{8}V_{2}V_{3}+c_{9}V_{3}^{2}+c_{10}V_{1}^{3}+c_{11}V_{1}^{2}V_{2}+c_{12}V_{1}^{2}V_{3}\\ \;+c_{13}V_{1}V_{2}^{2}+c_{14}V_{1}V_{2}V_{3}+c_{14}V_{1}V_{3}^{2}+c_{15}V_{2}^{3}+c_{16}V_{2}^{2}V_{3}+c_{17}V_{2}V_{3}^{2}+c_{18}V_{3}^{3}+\cdots +c_{55}V_{3}^{5} \end{array}$$

where *y* denotes the output of this model, which is *V*·cos *θ* or *V*·sin *θ*. Coefficients *c*_n_ were obtained by the least-squares method using the linear regression class in the Python sklearn module. The actually obtained coefficients fitted by the dataset are shown in the Supplementary Materials. This equation was approximated with a first-degree equation when the wind velocity was low. On the other hand, the Reynolds number of the sensor structure becomes less than 10^3^ below 1.0 m/s wind velocity. Then, the drag coefficient *C*_d_ approaches an inversely proportional relationship to the Reynolds number [24]. Since the pressure distribution has similar physical characteristics, the physical model *y* is approximated as

$$y\propto v^{2}\cdot C_{d}\sim v^{2}\frac{1}{Re}=v^{2}\frac{\nu }{v\cdot L}\propto v$$

where *ν* and *L* are kinematic viscosity and characteristic linear dimension. Thus, the equation matches the physical model around the device because it is also regarded as linear under low-velocity conditions.

**Figure 9:**
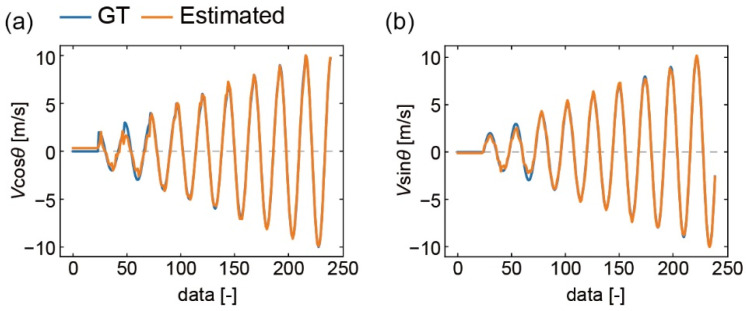
Results of converting the output of the three DP sensors to (**a**) *V*·cos *θ* and (**b**) *V*·sin *θ* using a regression model. “Data” refers to the data obtained by arranging the sensor voltage values from the wind tunnel experiment in ascending order of wind velocity, calculating the average of 30 measurements at the same angle within the same wind velocity, and arranging them in ascending order of the wind direction.

**Figure 10:**
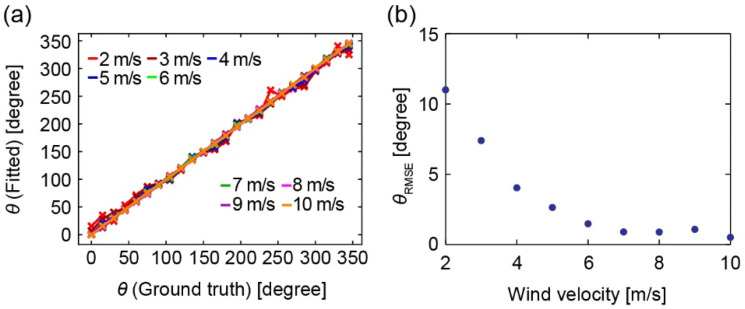
(**a**) Calculated wind direction and error. (**b**) Calculated *θ*_RMSE_ of the wind direction against the wind velocity.

The *V*·cos *θ* and *V*·sin *θ* calculated by the models are shown in Figure 9. This figure shows the values of Equation (1) output when $V_{1}$, $V_{2}$, and $V_{3}$ in the dataset were input. *V*·cos *θ*’s errors and *V*·sin *θ*’s errors are 0.33 m/s and 0.10 m/s, respectively, when the wind velocity is 0 m/s. This error means that the fitting error was less than this value. Both values shift from the GT at 2 and 3 m/s; however, they are consistent at larger wind velocities. The wind direction calculated by the model is shown in Figure 10a. In the low-velocity range, the value is slightly different from GT. We calculated the root mean square error (RMSE) of the wind direction, *θ*_RMSE_, for each wind velocity (Figure 10b). The *θ*_RMSE_ value is 11° at 2 m/s; however, it decreases as the velocity increases. This value eventually becomes 0.51° at 10 m/s. The smaller the output velocity is, the more unstable the airflow is in the wind tunnel test. Therefore, it is thought that *θ*_RMSE_ increases with decreasing wind velocity. Thus, it is thought that the model can be refined more accurately if more wind tunnel tests are performed in low-speed regions, including less than 2 m/s.

### 4.4. Drone Demonstration

**Figure 11:**
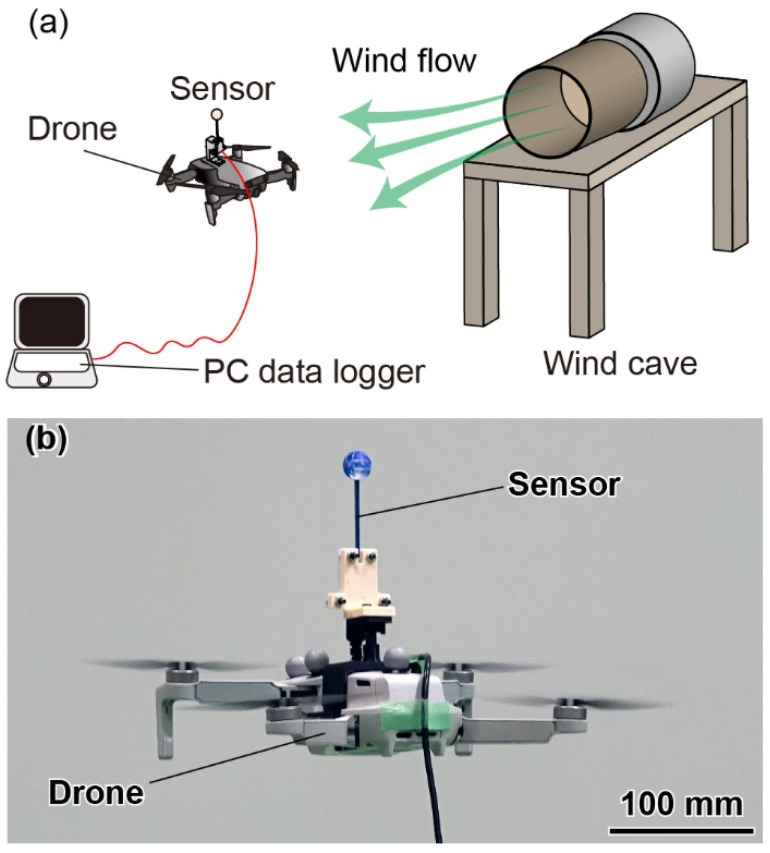
(**a**) Schematic diagram and (**b**) photograph of the experimental setup to evaluate the response when the sensor is mounted on a drone.

We conducted a drone flight test by attaching the fabricated airflow sensor to a toy drone (DJI Mini 2, DJI, Shenzhen, China), as shown in Figure 11. The sensor was fixed at approximately 130 mm above the fuselage with a jig to reduce the effects of propeller flow on the sensor response during the flight. The sensor signal was measured via a cable. The total weight of the sensor and jig was approximately 40 g, and the drone was capable of stable flight even with the attachments. The experiment was performed in an indoor space of 18 m × 9 m × 8 m. An airflow fan (SJF-300 L-1, Suiden, Gunma, Japan) was located at the center of the space, with a height of 1.7 m from the ground, where the ground effect of the adopted drone was negligible. The spatial airflow distribution was constructed by the linear interpolation of the flow velocities measured manually with an anemometer (Climomaster, Kanomax, Osaka, Japan) under the condition of stable fan operation. The four reflective markers were attached to the fuselage to obtain the 3D positions of the drone and sensor. The sensor position, flight speed and attitude were calculated using the 3D reconstructed points of the markers obtained from the multicamera motion-capture system (Optitrack, Corvallis, OR, USA) filmed at 120 Hz, which is sufficiently high compared with the drone flight speed. Then, the inflow velocities at the sensor were estimated. The drone was operated by a smartphone application (DJI FLY, DJI). First, the drone was moved from the starting ground to the front of the airflow fan. Then, the response was measured when the drone was rotated around the spot.

**Figure 12:**
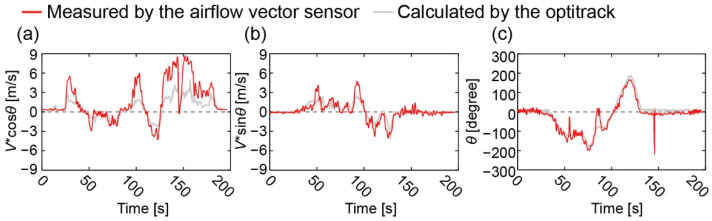
Measured (**a**) *V*·cos *θ*, (**b**) *V*·sin *θ*, and (**c**) *θ* during the drone flight.

The *V*·cos *θ*, *V*·sin *θ,* and *θ* calculated using the sensor output with the regression model are plotted in Figure 12 with red lines. The estimated values from camera images are also plotted in the same graphs with gray lines. There is a small phase lag between the two plots, indicating that the sensor time constant is significantly short. Additionally, we can see that *V*·sin *θ* had a smaller error than *V*·cos *θ*. Large differences in *V*·cos *θ* between the airflow sensor and the estimated values are observed in accordance with large positive values detected by the sensor, while good agreement is obtained in the range of small velocities during 40–90 s (Figure 12a). A large error was also observed in *V*·sin *θ* at approximately 50 and 90 s (Figure 12b). Such errors are thought to be mainly due to the induced flow generated by the flying drone itself. As a first step for the integrated drone and its stable flight, we set the developed sensor at approximately 130 mm from the fuselage; however, the higher position is expected to minimize these effects. In terms of wind direction, the graph shows excellent agreement most of the time (Figure 12c).

As mentioned in Section 4.3, if more wind tunnel tests are conducted to obtain the dataset for the regression modeling, then the error in wind velocity is expected to be reduced in low-speed regions so that the sensor reliability will be improved to a greater extent. Additionally, there was nonmonotonic behavior in the relationship between the sensor output and wind direction, as shown in Figure 8b. This nonmonotonic behavior can be attributed to the pressure distribution acting on the sphere surface under laminar flow with Reynolds number around 10^3^~10^4^, as described in Section 2.2. By utilizing multiple inlets for each DP sensor channel, this nonmonotonic behavior would be reduced [14] so that the sensor robustness would be further enhanced.

## 5. Conclusions

We designed, fabricated, and evaluated a compact and lightweight sphere-shaped airflow vector sensor onboard a drone. The proposed sensor consisted of hemispherical housings and a substrate with MEMS DP sensors. Three pairs of inlets were formed on the spherical surface, and each DP sensor detected the DP applied to the corresponding pair of inlets. Since the pairs of inlets were formed at 60° intervals, the 2D wind velocity and direction were measured with high robustness. The fabricated sensor housing was 16 mm in diameter, and the total weight, including the substrate, was 4.4 g. The sensor output was calibrated by a wind tunnel test in the range of 2–10 m/s with changing inflow angle. It was confirmed that the regression models with the three DP sensor outputs were able to derive the wind velocity and direction. Finally, it was demonstrated that the fabricated sensor was mounted on a toy drone so that the wind velocity and direction of the artificial crosswind were measured during flight. By further expanding the dataset and improving the regression model, it is expected that the proposed sensor can be utilized for monitoring air current during a drone flight.

## Data Availability

The data presented in this study are available on request from the corresponding author.

## Supplementary Materials

The following supporting information can be downloaded at: https://www.mdpi.com/article/10.3390/s22031087/s1, Table S1: Coefficients of Equation (1); Figure S1: Responses of the DP sensor of inlet 3 against step responsive airflow.

## References

[1] Scott J.E., Scott C.H. Drone delivery models for healthcare. Proceedings of the 50th Hawaii International Conference on System Sciences.

[2] Kim S.J., Lim G.J., Cho J., Côté M.J. (2017). Drone-Aided Healthcare Services for Patients with Chronic Diseases in Rural Areas. J. Intell. Robot. Syst. Theory Appl..

[3] Namani S., Gonen B. Smart agriculture based on IoT and cloud computing. Proceedings of the 2020 3rd International Conference on Information and Computer Technologies (ICICT).

[4] Schofield O.B., Lorenzen K.H., Ebeid E. Cloud to Cable: A Drone Framework for Autonomous Power line Inspection. Proceedings of the 2020 23rd Euromicro Conference on Digital System Design (DSD).

[5] Kalaitzakis M., Vitzilaios N., Rizos D.C., Sutton M.A. (2021). Drone-Based StereoDIC: System Development, Experimental Validation and Infrastructure Application. Exp. Mech..

[6] Robinson T.R. (1869). On a New Anemometer.

[7] Kim S., Kubicek R., Paris A., Tagliabue A., How J.P., Bergbreiter S. A whisker-inspired fin sensor for multi-directional airflow sensing. Proceedings of the 2020 IEEE/RSJ International Conference on Intelligent Robots and Systems (IROS).

[8] Lynnworth L.C., Liu Y. (2006). Ultrasonic flowmeters: Half-century progress report, 1955–2005. Ultrasonics.

[9] Arens E., Ghahramani A., Przybyla R., Andersen M., Min S., Peffer T., Raftery P., Zhu M., Luu V., Zhang H. (2020). Measuring 3D indoor air velocity via an inexpensive low-power ultrasonic anemometer. Energy Build..

[10] Anemometry H., Comte-bellot G. (1976). Hot-wire anemometry. Annu. Rev. Fluid Mech..

[11] Sadeghi M.M., Peterson R.L., Najafi K. (2013). Air flow sensing using micro-wire-bonded hair-like hot-wire anemometry. J. Micromech. Microeng..

[12] Klopfenstein R. (1998). Air velocity and flow measurement using a Pitot tube. ISA Trans..

[13] Liu C., Du L., Zhao Z., Fang Z., Li L. A directional anemometer based on MEMS differential pressure sensors. Proceedings of the The 9th IEEE International Conference on Nano/Micro Engineered and Molecular Systems (NEMS).

[14] Bruschi P., Dei M., Piotto M. (2009). A low-power 2-D wind sensor based on integrated flow meters. IEEE Sens. J..

[15] Piotto M., Pennelli G., Bruschi P. (2011). Fabrication and characterization of a directional anemometer based on a single chip MEMS flow sensor. Microelectron. Eng..

[16] Bruschi P., Piotto M., Dell’Agnello F., Ware J., Roy N. (2016). Wind Speed and Direction Detection by Means of Solid-state Anemometers Embedded on Small Quadcopters. Procedia Eng..

[17] Minh-Dung N., Takahashi H., Kuwana K., Takahata T., Matsumoto K., Shimoyama I. 3D airflow velocity vector sensor. Proceedings of the 2011 IEEE 24th International Conference on Micro Electro Mechanical Systems.

[18] Eckman R.M., Dobosy R.J., Auble D.L., Strong T.W., Crawford T.L. (2007). A pressure-sphere anemometer for measuring turbulence and fluxes in hurricanes. J. Atmos. Ocean. Technol..

[19] Jing X.M., Lu J.Y., Miao J.M., Hans H., Rahman H.A., Pan S.S., Norford L., Centre T.S. (2011). Hot-Film Sensors for 2-D Environmental Airflow Monitoring.

[20] Gao S., Yi Z., Ye Y., Qin M., Huang Q.A. (2019). Configuration of a Self-Heated Double Wheatstone Bridge for 2-D Wind Sensors. J. Microelectromech. Syst..

[21] Zhu Y.Q., Chen B., Qin M., Huang J.Q., Huang Q.A. (2015). Development of a self-packaged 2D MEMS thermal wind sensor for low power applications. J. Micromech. Microeng..

[22] Ye Y., Yi Z., Gao S., Qin M., Huang Q.A. (2018). Octagon-Shaped 2-D Micromachined Thermal Wind Sensor for High-Accurate Applications. J. Microelectromech. Syst..

[23] Leoni A., Barile G., Muttillo M., Pantoli L., Stornelli V., Ferri G., Paolucci R., Vita L. (2017). Di A Spherical Directional Anemometer Sensor System. Proceedings.

[24] Hoerner S.H. (1965). Fluid—Dynamic Drag.

[25] Takahashi H., Dung N.M., Matsumoto K., Shimoyama I. (2012). Differential pressure sensor using a piezoresistive cantilever. J. Micromech. Microeng..

[26] Takahashi H., Nakai A., Shimoyama I. (2018). Waterproof airflow sensor for seabird bio-logging using a highly sensitive differential pressure sensor and nano-hole array. Sens. Actuators A Phys..

[27] Takahashi H., Naruoka M., Inada Y., Sato K. (2021). Seabird biologging system with compact waterproof airflow sensor. J. Robot. Mechatron..

