# Supplementary material for: Compact Sphere-Shaped Airflow Vector Sensor Based on MEMS Differential Pressure Sensors
Source: Sensors (Basel). 2022 Jan 30;22(3):1087. doi: 10.3390/s22031087 (PMC8838244; doi:10.3390/s22031087)
Supplement: Supplementary file 1 [file sensors-22-01087-s001.zip › sensors-1533317-supplementary.pdf]

Supplementary Material for Compact Sphere-Shaped Airflow Vector Sensor Based on MEMS Differential Pressure Sensors

Kotaro Haneda [1], Kenei Matsudaira [1], Ryusuke Noda [2], Toshiyuki Nakata [3], Satoshi Suzuki [3], Hao Liu [3] and Hidetoshi Takahashi [1,*]

[1] Department of Mechanical Engineering, Faculty of Science and Technology, Keio University, 3-14-1 Hiyoshi, Kouhoku-ku, Yokohama 223-8522, Japan; hane_taro@keio.jp (K.H.); kenei.matsudaira@takahashi.mech.keio.ac.jp (K.M.)
[2] Department of Aeronautics and Astronautics, Graduate School of Engineering, Kyoto University, Kyoto Daigaku-Katsura, Nishikyo-ku, Kyoto 615-8540, Japan; noda.ryusuke.6a@kyoto-u.ac.jp
[3] Graduate School of Engineering, Chiba University, 1-33 Yayoi-cho, Inage-ku, Chiba 263-8522, Japan; tnakata@chiba-u.jp (T.N.); suzuki-s@chiba-u.jp (S.S.); hliu@faculty.chiba-u.jp (H.L.)
* Correspondence: htakahashi@mech.keio.ac.jp; Tel.: +81-45-566-1847

Table S1. Coefficients of Equation (1).

$$y = c_0 + c_1V_1 + c_2V_2 + c_3V_3 + c_4V_1^2 + c_5V_1V_2 + c_6V_1V_3 + c_7V_2^2 + c_8V_2V_3 + c_9V_3^2 + c_{10}V_1^3 + c_{11}V_1^2V_2 + c_{12}V_1^2V_3 + c_{13}V_1V_2^2 + c_{14}V_1V_2V_3 + c_{14}V_1V_3^2 + c_{15}V_2^3 + c_{16}V_2^2V_3 + c_{17}V_2V_3^2 + c_{18}V_3^3 + \cdots + c_{55}V_3^5 \quad (1)$$

(**a**) Coefficients for V·cosθ

c_0	c_1	c_2	c_3	c_4	c_5	c_6	c_7	c_8	c_9
-3.46E+06	-1.16E+03	-1.89E+03	-7.68E+02	-1.52E+04	5.16E+04	2.23E+04	-5.16E+04	6.72E+04	-2.02E+04
c_{10}	c_{11}	c_{12}	c_{13}	c_{14}	c_{15}	c_{16}	c_{17}	c_{18}	c_{19}
1.55E+07	9.73E+07	1.69E+07	-1.10E+07	2.68E+06	2.40E+07	1.62E+07	1.26E+07	5.91E+07	2.21E+06
c_{20}	c_{21}	c_{22}	c_{23}	c_{24}	c_{25}	c_{26}	c_{27}	c_{28}	c_{29}
-1.80E+08	5.31E+09	-1.58E+10	-1.05E+10	-6.75E+08	-1.73E+10	1.15E+08	-5.67E+09	1.54E+10	-4.07E+09
c_{30}	c_{31}	c_{32}	c_{33}	c_{34}	c_{35}	c_{36}	c_{37}	c_{38}	c_{39}
4.26E+08	-2.63E+09	1.14E+09	-8.10E+08	2.42E+08	-2.45E+10	-4.18E+12	2.76E+12	5.50E+12	-1.59E+12
c_{40}	c_{41}	c_{42}	c_{43}	c_{44}	c_{45}	c_{46}	c_{47}	c_{48}	c_{49}
4.34E+12	-5.12E+12	1.72E+12	4.25E+12	6.05E+11	2.34E+12	1.35E+12	-1.05E+13	5.61E+11	-7.19E+11
c_{50}	c_{51}	c_{52}	c_{53}	c_{54}	c_{55}				
-2.88E+11	-1.18E+11	-2.75E+11	-7.95E+11	-2.69E+10	-3.76E+09				

(**b**) Coefficients for V·sinθ

c_0	c_1	c_2	c_3	c_4	c_5	c_6	c_7	c_8	c_9
-3.28E+06	1.63E+03	-6.35E+01	-1.45E+03	5.86E+04	-6.65E+03	-5.74E+04	-7.57E+03	8.94E+04	-3.40E+04
c_{10}	c_{11}	c_{12}	c_{13}	c_{14}	c_{15}	c_{16}	c_{17}	c_{18}	c_{19}
-1.24E+07	-1.59E+07	5.35E+07	-8.91E+06	1.13E+07	-3.14E+07	-4.44E+06	1.04E+08	-3.90E+07	7.70E+06
c_{20}	c_{21}	c_{22}	c_{23}	c_{24}	c_{25}	c_{26}	c_{27}	c_{28}	c_{29}
-5.67E+08	-7.41E+09	9.68E+09	7.60E+09	3.50E+09	-2.37E+09	3.67E+09	1.24E+10	-5.81E+09	-2.18E+09
c_{30}	c_{31}	c_{32}	c_{33}	c_{34}	c_{35}	c_{36}	c_{37}	c_{38}	c_{39}
-9.17E+08	-5.51E+09	5.65E+09	-1.53E+09	4.08E+08	1.37E+11	-2.16E+12	1.70E+12	7.98E+12	1.61E+12
c_{40}	c_{41}	c_{42}	c_{43}	c_{44}	c_{45}	c_{46}	c_{47}	c_{48}	c_{49}
-2.78E+12	-7.40E+12	-1.83E+13	1.33E+13	-4.91E+12	1.64E+12	9.97E+12	-3.89E+13	1.67E+13	-5.34E+10
c_{50}	c_{51}	c_{52}	c_{53}	c_{54}	c_{55}				
-1.10E+11	-5.58E+12	1.24E+13	-8.84E+12	1.66E+12	-4.06E+10				

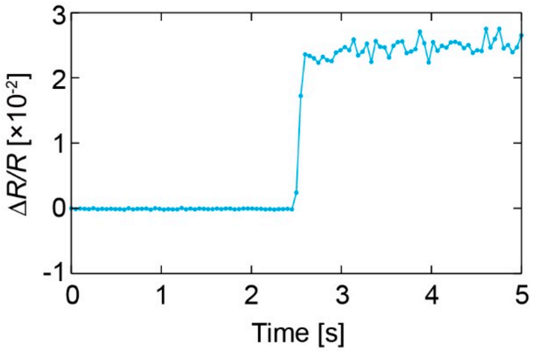

Figure S1. Responses of the DP sensor of inlet 3 against step responsive airflow.
